# Telehealth and Precision Prevention: Bridging the Gap for Individualised Health Strategies

**DOI:** 10.1055/s-0044-1800720

**Published:** 2025-04-08

**Authors:** Edwin Chi Ho Lau, Vije Kumar Rajput, Inga Hunter, Jose F. Florez-Arango, Prasad Ranatunga, Klaus D. Veil, Gumindu Kulatunga, Shashi Gogia, Craig Kuziemsky, Marcia Ito, Usman Iqbal, Sheila John, Sriram Iyengar, Anandhi Ramachandran, Arindam Basu

**Affiliations:** 1 The Hong Kong Hospital Authority; 2 London School of Hygiene and Tropical Medicine; 3 Massey University; 4 Weill Cornell Medicine Department of Population Health Science; 5 Provincial Department of Health Services, North-Western Province, Sri Lanka; 6 Western Sydney University; 7 Health Information Unit, Ministry of Health, Sri Lanka; 8 Society for Administration of Telemedicine and Health Care Informatics; 9 MacEwan University; 10 Unidade de Pós-Graduação, Extensão e Pesquisa do Centro Paula Souza; 11 School of Population Health, Faculty of Medicine and Health, University Of New South Wales, Sydney, Australia; 12 Teleophthalmology and E-learning departments, Sankara Nethralaya, Chennai, India; 13 Department of Internal Medicine, University of Arizona College of Medicine; 14 Department of Health Information Technology, International Institute of Health Management Research, Delhi, India; 15 University of Canterbury

**Keywords:** Precision, Prevention, Telehealth

## Abstract

**Introduction** : Precision prevention has shown an upsurge in popularity among epidemiologists in both developed and developing countries in the past decade.

**Objectives** : Initially practiced in oncology, this approach is increasingly adopted in public health to guard against other common non-communicable diseases (NCDs), such as diabetes and cardiovascular diseases. It aims to tailor preventive measures according to each individual's unique characteristics, such as genomic data, socio-demographic features, environmental factors, and cultural background.

**Methods** : Healthcare information technologies, including telehealth and artificial intelligence (AI), have served as a vital catalyst in the expansion of this field in the past decade. Under this framework, real-time contemporaneous clinical data is collected via a wide range of digital health devices, such as telehealth monitors, wearables, etc., and then analyzed by AI or non-AI prediction models, which then generate preventive recommendations.

**Results** : The utilization of telehealth technologies in the precision prevention of cardiovascular diseases (CVDs) is a very illustrative application. This paper explores these topics as well as certain limitations and unintended consequences (UICs) and outlines telehealth as a core enabler of precision prevention as well as public health.

## 1. Introduction

The importance of disease prevention in a tiered fashion, rather than reactive treatment, is especially true for non-communicable diseases (NCDs), including cancers and other chronic medical illnesses, which are often long-term and impose significant societal and economic burdens on developed and developing nations alike [ 1 ]. Precision medicine is about connecting people to tailored, personalized health services. It moves away from a one-size-fits-all model to a more personalized model. In fact, the concept of providing individual-based medical care has long been practiced among different cultures in both the East and the West.

There are many ways that digital health can contribute to the delivery of precision medicine, including approaches and tools such as artificial intelligence, electronic health records, and big data [ 2 ]. Digital health can support precision medicine by enabling ongoing data collection and patient monitoring to tailor a patient's health and wellness plan to their needs. Saban-Nejad et al. (2020) provided a list of seven pillars that were needed to support precision digital health and medicine: precision observation and assessment, precision health promotion, precision engagement, precision early detection, precision prevention, precision treatment, as well as precision equity [ 3 ].

Common to all precision health delivery frameworks is the need for both structural and behavioral support. Enabling data collection to support ongoing patient monitoring and observation will only be as good as the level of reinforcement and empowerment that patients and healthcare providers receive from their structural environment. Besides technology, the pursuit of precision public health must also consider managing change in the health beliefs and behaviors of both patients and healthcare workers. In other words, technology, including telemedicine, mainly serves as a tool to facilitate bidirectional data collection, analysis and transfer to achieve the goal of personalized care in society, which depends on collaborative efforts from healthcare practitioners, public health researchers, policymakers and the general public. Previous work from our Telehealth Workgroup (THWG) has highlighted how telehealth can support systems-based health transformation at the individual and public health levels [ 4 ]. Kuziemsky et al. (2019) have argued that by combining artificial intelligence with telemedicine, new models of patient care are created, and we agree that the same concept can be adopted to develop new personalized models in preventive medicine. We extend that work in this paper by describing how telehealth can provide specific digital health support for precision prevention.

In this position paper, we begin with the concept and definition of precision prevention, followed by describing the evolving approaches of digital health in delivering precision prevention. We then describe the specific roles of telehealth in this field, supported by an illustrative example of cardiovascular disease prevention. Finally, we discuss the implications of our work, including limitations and unintended consequences of telehealth-enabled precision prevention.

## 2. Precision Prevention - adapting Prevention for the Digital Health age

Traditionally, preventive medicine was divided into universal population-based and high-risk prevention strategies. Their respective strengths and weaknesses were well addressed by Professor Geoffery Rose in two of his landmark publications in the 1980s, generating the well-recognized phenomenon of the “Prevention Paradox” [ 5 ] (Rose, 1985). According to his theory, population-based strategies targeting the general population tend to benefit the population as a whole rather than individuals; on the other hand, high-risk strategies tend to identify and treat individuals with the highest risk of disease, resulting in more benefits at the individual level [ 6 ]

Precision prevention, a special form of precision medicine, appears to strike a balance between the two extremes. It is broadly defined as “the customization of disease preventive strategies to specific individuals or populations, based on a constellation of biologic, behavioral, socioeconomic and epidemiological data” [ 7 ]. It is based on the premise that no two persons are alike, and thus, the delivery of health care should be tailored to everyone's unique characteristics [ 8 ]. As a result, screening and detection can be performed more effectively, minimizing risky, unnecessary procedures. As logical as it sounds, it is a relatively new term, first appearing in an editorial about cancer prevention in 2014 [ 9 ].

In 2015, US President Barack Obama proposed the Precision Medicine Initiative, which served as the blueprint of a broader state-wide research program that aimed to build the essential evidence base for precision medicine [ 10 ]. Although genomic medicine was the primary focus at that time, it should be reiterated that these two terms, genomic medicine and precision medicine, are not interchangeable. This fundamental principle is often overlooked even at the professional level; for example, the American Cancer Society still describes precision medicine as “a way health care providers can offer and plan specific care for their patients, based on the particular genes, proteins and other substances in a person's body”.

The foundation of any precision prevention framework lies in the concept of “P4 medicine, which represents predictive, personalized, preventive, and participatory medicines respectively. In this concept, one's health status is estimated based on a constellation of macro- or micro-level risk factors including biological, socio-demographic or cultural characteristics (“predictive”); then the predicted risk is used to guide subsequent tailored measures (“personalized”) aiming at preventing potential adverse health consequences (“preventive”), which depends on the joint efforts and contributions from patients, clinicians, and the entire healthcare community (“participatory”) [ 11 ]. The key objective is to detect at-risk individuals earlier so that they may benefit from earlier interventions.

The earliest framework for precision prevention was proposed by Rebbeck on the prevention of cancer, which consisted of the following six major components [ 9 ]:

- Identify the main risk factors of the disease as per the tiered risk model, i.e. universal, selected, and indicated population risk groups;
- Understand the socio-behavioral factors that modulate the effectiveness of screening;
- Identify the “communities of need” that may benefit from precision prevention strategies, so that they may be engaged early in the conceptualization, formulation, and implementation of such strategies;
- Develop efficacious primary, secondary, and tertiary prevention interventions;
- Engage the relevant stakeholders in accepting, implementing and sustaining the above strategies - the general public, patients, healthcare professionals, policymakers etc;
- Evaluate the impact of the program through community-engaged research, geospatial research and health services, systems analyses etc.

In the past ten years, this framework was expanded in subsequent publications to include other non-communicable diseases besides cancer [ 12 ].

## 3. The Evolution of Digital Technologies in Precision Prevention

Despite its indisputable benefits in public health, this multidimensional approach of precision prevention is yet to be widely adopted, mainly because of the difficulties in capturing, processing, and interpreting an enormous dataset [ 13 ]. Nonetheless, digitalization in the modern era has aroused the interest of public health researchers in exploring the use of digital technologies in preventive medicine [ 14 ].

Current precision prevention approaches mainly focus on the previous or current health behaviors of an individual, achieving personalization according to discrete subgroup characteristics. Statistical risk prediction models were built upon datasets from retrospective population-based observational studies or prospective cohort studies based on standardized or harmonized state registry data. A noticeable example is the Nurses' Health Studies, an ongoing series of cohort studies since 1976 that have recruited more than 280,000 participants so far and investigate the risk factors of a number of chronic diseases [ 15 ]. According to a recent scoping review on behavioral change interventions, the majority of personalized interventions are still designed manually by clinicians, and the mode of delivery is mostly via human interactions, either through telephone calls or face-to-face interviews [ 16 ].

Nonetheless, process mining is currently underway to develop the appropriate clinical pathways and protocols for utilizing new technologies in this regard [ 17 , 18 ]. For instance, artificial intelligence (AI) has been proposed to support primary, secondary, and tertiary prevention strategies by enabling population screening, tailoring the management of diseases and minimizing complications of chronic diseases.

With the rapid upsurge of big data, cloud computing, AI, and the Internet of Things (IoT), a revolutionary approach to precision health, the “Digital Twin” (DT) has caught the attention of researchers, clinicians, and epidemiologists. It is a digital replica of a physical entity, and the digital and the physical twins are interconnected with real time data that change the state of each other [ 19 ]. In the healthcare sector, human digital twins allow healthcare professionals to gather, integrate, analyze, and interpret a wealth of individualized data from various contemporaneous sources of real-world evidence (RWE), such as digital wearables, implantables, smart home devices, mobile health applications, electronic medical records [ 12 ]. Using machine learning and longitudinal data, digital twins can generate predictive models to forecast disease progression throughout the whole life course of an individual. Thus, it can be envisaged that digital health may contribute to precision health in many ways, and in the same line of thought, we believe that telemedicine could be an important resource for the adoption of precision prevention, as we explain in the next section.

## 4. Telehealth as an Essential Component in Precision Prevention

Telehealth, or telemedicine, is defined as the use of information and telecommunication technologies to transfer medical information for the delivery of clinical and educational services, across time and/ or distance. It can be classified as synchronous using real-time electronic communication, asynchronous using store-and-forward communication, or remote monitoring involving data collection through distributed devices using the Internet of Things (IoT) [ 4 ]. In the field of precision prevention, telehealth is essential to bridge the gap between patients and healthcare service providers, beginning at the stage of data acquisition and continuing through the delivery of interventions. It can be integrated into existing precision prevention frameworks to achieve both disease control and risk factor control at the primary, secondary, and tertiary levels.

At the stage of data collection, telehealth facilitates the acquisition of multidimensional data with people at the epicenter, such as biological data, medical records, socio-demographic characteristics and psychological profile [ 12 ]. Conventionally, these data are fixed and scattered, often captured as point prevalence snapshots. Telehealth enables the safe capture and protected transmission of an enormous amount of data, both synchronously or asynchronously. This is particularly useful in primary prevention, where the response rate using traditional information-gathering methods (e.g., health check-ups, questionnaires, interviews) is notoriously low. Regarding secondary or tertiary prevention, telehealth can also facilitate the gathering of information such as symptoms or personal feelings by means of teleconsultations among clinicians, patients, and even their families and friends, overcoming logistic or geographical barriers [ 16 ].

When it comes to intervention delivery, the role of telehealth is twofold. First, many preventive interventions, especially behavioral advice or lifestyle coaching, can be delivered efficiently through mobile applications (mHealth), electronic platforms (eHealth), or virtual teleconsultations, saving the time and cost incurred in physical attendance and traveling. Coordination among disciplines is then optimized, minimizing resource wastage due to fragmented or overlapping services [ 20 ]. Secondly, individuals are empowered to generate, interpret, and monitor their own health data, which, when back-transmitted to the central data registry, would in turn form a feedback loop and contribute to the refinement of prediction models [ 21 ].

In summary, the use of telehealth may benefit precision prevention at both individual and population levels. For individuals, telehealth does not only help with direct patient management and follow-up but also allows the simultaneous collection and transmission of health data (medical conditions, behavioral, socio-demographic and environmental characteristics, etc.), which provides valuable input to prognosis estimation and clinical management plan. At the population level, the combination of telehealth and precision prevention frameworks can strengthen the coordination among disciplines in health service delivery such that health resource allocation can be optimized. From a public health perspective, risk factors of chronic diseases at all tiers (primary, secondary, and tertiary) can be addressed precisely and adaptively, which provides invaluable guidance in health budget and strategic planning.

## 5. A Practical Example - Precision Prevention in Cardiovascular Diseases (CVDs)

Cardiovascular diseases (CVDs) impose a huge burden on both developed and developing countries alike. As such, the use of telehealth in the precision prevention of CVDs has gained much popularity among medical practitioners, researchers, and patients. Using this common non-communicable disease as an example, we would highlight the role and significance of telehealth in terms of disease control and risk factor control.

### 5.1. Disease control

Teleconsultation. The COVID-19 pandemic has revealed the importance of remote healthcare in times of social distancing and geographical separation. Telemedicine ensured the continued delivery of specialist care to patients with vulnerable disease states, such as advanced heart failure, despite logistic and geographical barriers [ 22 ]. This is important for continued monitoring of disease progress and prevention of complications. Studies have shown that continuity of care in patients with heart failure was negatively impacted during the COVID-19 pandemic because of the social distancing measures and redistribution of healthcare resources, and that telemedicine was an effective alternative to direct patient care delivery [ 23 ].

Telepharmacy. As important as specialist medical care in the management of cardiovascular disease is medication adherence. Telepharmacy ensures the continued dispensing of medications, provision of pharmaceutical advice, and monitoring of drug compliance and side effects. Through remote medication therapy management, tele-pharmacists may help to ensure that patients are adhering to their medication regimens and can counsel patients should they have any questions [ 24 ].

Digital rehabilitation program. Multidisciplinary cardiac rehabilitation programs are pivotal in the tertiary prevention of patients with CVDs. In a randomized controlled trial comparing the effect of a digital cardiac rehabilitation program delivered online and standard practice, it was found that the incorporation of digital health interventions in the rehabilitation program led to a significant reduction in body weight, as well as cardiovascular-related emergency visits and hospitalizations in patients suffering from acute coronary syndromes (ACS) after percutaneous coronary intervention (PCI) [ 25 ]. This gave strong evidence that the utilization of digital platforms in the provision of rehabilitation care resulted in better outcomes. The provision of multidisciplinary care service to patients also improves coordination and communication among different healthcare professionals, reducing unnecessary resource wastage and duplicated care.

### 5.2. Risk factor control

Physiological monitoring. Digital wearables (e.g. smartwatches, accelerometers) provide an excellent means for patients and healthcare providers to keep track of physiological parameters, such as heart rate, blood pressure, physical activity level, oxygen level etc., ideally automated and real-time. A large-scale meta-analysis involving more than 160,000 participants showed that the use of wearable activity trackers resulted in a significant improvement in physical activity, body composition and fitness, which translated to approximately 1,800 extra steps per day, 40 min per day more walking, and 1 kg reduction of weight [ 26 ]. This gave evidence favoring the use of activity trackers in monitoring disease activity.

Remote weight reduction program. Since obesity is one of the major determining factors in the prevalence and prognosis of patients with CVDs, efforts have been made to explore the effectiveness of using telemedicine technologies in this regard. In a randomized controlled trial involving 223 patients, it was found that patients who received a tailored online coaching program on physical activity (CardioFit) had a significantly higher objectively measured (p = 0.023) and self-reported physical activity (p = 0.047) compared to usual care. Emotional (p = 0.038) and physical (p = 0.031) dimensions of heart disease health-related quality of life were also higher with CardioFit [ 27 ]. Another randomized controlled trial recruiting 415 obese patients and at least 1 CVD risk factor showed a significant and sustained weight loss for 24 months with the use of remote personalized weight reduction support [ 28 ].

## 6. Discussion

The use of telehealth in precision prevention, just like other digital health initiatives, is a double-edged sword. The benefits mentioned in the case example above could be accompanied by a wide range of emerging challenges and unintended consequences (UICs), including those described in our 2016 yearbook paper [ 29 ]. As such, instead of repeating known issues, we would like to highlight the key issues relevant to our theme of precision prevention. These issues can be broadly divided into five main areas: 1) limitation in care delivery; 2) behavioral modifications of both patients and providers; 3) equity and ethical concerns; 4) data privacy and cybersecurity concerns; and 5) societal and financial impact.

*Limitations in Care Delivery.* No matter how advanced telemedicine has become, direct physical examination between patient and the remote clinician is inherently impossible in virtual care. As a result, important information required to make clinical judgments might not be fully available to clinicians, causing misinterpretation, wrong diagnosis, and wrong recommendations. This is the reason telemedicine is meant to augment, rather than replace, in-person care delivery.

Another significant issue is “e-iatrogenesis”, which can be defined as “patient's harm resulting from the application of health information technologies” [ 30 ]. The use of AI in data analysis must consider the statistical power of the machine learning model, as well as the clinical significance of the results, which may only represent normal variants instead of genuine anomalies. Unawareness of this pitfall might lead to over-investigations and unnecessary side effects from investigations. In addition, the reliability, robustness, and safety of AI systems must also be addressed, since the monitoring of complex machine learning systems is often very difficult if not impossible.

*Behavioral Modifications.* As human behaviors are complex and dynamic with a high level of individual variability, telehealth may bring unintended modification of behaviors. From the patient's perspective, a phenomenon called “trained helplessness” may arise, meaning if a well-adopted technology which drives a behavioral change is suspended for any reason, subjects may revert to their old lifestyle behaviors. A classic example is medication adherence reminders such as smart pill dispensers. Moreover, subjects may become so obsessed with self-monitoring that anxiety or hypochondria may ensue [ 31 ]. From the clinician's perspective, over-reliance on virtual care might lead to the deskilling of their physical examination or manual procedural skills, let alone the new skill sets that they must acquire to provide a high-quality telecare service. All these may paradoxically lead to a higher rate of medical errors [ 29 ].

*Equity and Ethical Concerns.* Utilization of telemedicine and AI in precision prevention may introduce or exacerbate inequity between the haves and have-nots, between young and older generations, and between the digitally literate and illiterate populations [ 32 ]. Care must be taken to ensure that telemedicine is available to the rural, disabled or profoundly sick populations, whose access to healthcare facilities is most critical.

*Data Privacy and Cybersecurity Concerns.* The ease of data transmission, handling, and storage by means of networked medical devices and cloud services means that personal data is more vulnerable to cybersecurity issues such as unauthorized use or system hacking [ 33 ]. Various state-wide governance bodies are responsible for regulating and monitoring the data security standards of wearables, network infrastructures as well as cloud services. These include the US Food and Drug Administration, Cloud Security Alliance, as well as the Health Insurance Portability and Accountability Act (HIPAA) in the United States [ 34 ]. Care must be taken to strike a balance between individual privacy and public benefit.

*Societal and Financial Impact.* The rise of precision prevention invariably impacts individuals' insurance premiums as more risk factors are detected by means of digital wearables. Insurance companies might then manipulate precision prevention programs to maximize their profits. eHealth implementation might also disrupt existing health service payment reimbursement models as the distinction between formal consultations and informal conversations becomes more blurred [ 35 ]. At the public finance level, government investments in healthcare technologies, especially AI, are expected to increase, which would further complicate the overall taxation and public funding mechanism.

*Quality Control of Devices.* Remote capture and monitoring of individual physiological parameters are done via a wide variety of devices, which are often heterogeneous. Monitoring of the quality and standards of these devices is paramount to ensure that the information obtained is credible, valid, and interoperable for sound decision-making [ 36 ]. However, there are so far no universally accepted standards for these devices, and proper governance is lacking in many countries.

In summary, digital health technologies, including telehealth, play a major role in revolutionizing prevention medicine and enable the employment of individualized biological, sociodemographic and psychological factors in tailoring primary, secondary, and tertiary prevention strategies to the specific needs of individuals. The benefits of telehealth in this regard span from physiological data collection and provision of consultations, to delivery of behavioral interventions and monitoring of disease progress. Although there are some accompanying unintended consequences, collaborative efforts among healthcare providers, consumers, and academics should continue to explore its use in a wider perspective.
